# Prevention and Cure
*Three pounds six ounces weight (avoirdupois) of Testimonials to Candidates for the appointment of Medical Officer of Health. Contributed by various authors and authoresses. London: 1856.


**Published:** 1856-04

**Authors:** 


### PREVENTION AND CURE.*

Since the commencement of the modern period, when hygienic medicine began to undergo a revival after a slumber of seventeen hundred years, it has been an absurd practice to look upon the principles of prevention and cure as rival systems; not possibly as systems absolutely antagonistic, but as requiring, at least for their special advancement, distinct orders of intellect, education, and research.

This fallacy, which, even to the present moment, is firmly rooted in the majority of minds, is highly injurious both to the science of medicine and to the world at large. To medicine, because it makes a division where all should be one; to the world, because they who treat its diseases without being fully acquainted with the general principles of prevention, cannot know more than half their business; while they who undertake to prevent disease in the mass, without knowing intimately the nature of that which is to be prevented, are, of necessity, pretenders of no ordinary kind.

To understand clearly the reason of the distinctions which exist between the systems of preventive and curative medicine, it is necessary to take a comprehensive review of medical science as a whole, and of its position at various times in the intellectual world, since the success of each system belongs, to a certain extent, to a different phase of progress and civilisation.

The science of hygiene is a political as well as a medical study: it appeals to the mind universal; when it once takes root, it speedily indoctrinates all with its teachings; it models itself into a household truth, and commingles with the social, the moral, and even the religious elements of the mind. Admitted but briefly within the threshold of the citizen, it steps forth to take high place in the legislature, and becomes recognised eventually as a governing principle or law, against which to speak is to rave at common sense, and ignore the first feelings of self-preservation.

The curative system of medicine, on the other hand, has, as it is generally understood, a more exclusive meaning, being based mainly on the labours and knowledge of a distinct body of men, educated to its work. In a strict and

* Three pounds six ounces weight (avoirdupois) of Testimonials to Candidates for the appointment of Medical Officer of Health. Contributed by various authors and authoresses. London: 1856.

abstract sense, it assumes in some degree the non-necessity of prevention, since it claims to itself the power of acting as a safety valve to the world's indiscretions, ignorance, and frailties. Viewed in this light, it has been often held up to the gaze of human kind in the character of a life-restoring genius, and in many other poetical representations.

The times when the two systems here referred to have stood out most prominently in their isolated forms, are those when the educational status of the world has been at opposite points. The doctrine of absolute faith in the principle of disease prevention, indicates the existence of a high order of thought; of broad views of life and health, of diseases and their origins, of death and its meaning as a phenomenon of nature. The doctrine of absolute faith in curative medicine, vested in the hands of a separate sect or class, and exercised by them as by regal right, and without the assistance or interference of those upon whom it is exercised, indicates a lower standard of general knowledge; a too confiding spirit on the wisdom of a minority; a departure too wide from the law of self-preservation; an ignorance of the general causes of those evils which it is desirable to avoid; a blindness and therefore an unnecessary exposure to danger; an overweening and sudden fear at obvious dangers of all kinds, little and great; a hasty and thoughtless pursuit after that mode of rescue from disease which claims to itself the highest pretensions, and boasts, whether justly or not, of the largest successes.

The history of every age brings out in bold relief the truth of the statements here made. When old Rome in the midday of her life sought to avert the miseries of plague by the draught of the slave *medicus*, or the charm of the soothsayer, she presented the spectacle of a semi-cultivated nation, pinning its faith to an assumed curative system. When, in a later age, she sought to prevent these catastrophes by calling to her aid the mechanical genius of Vitruvius, and the medical learning of Celsus, she appeared in a second and advanced phase of civilization. And that which is thus true of Rome is equally true in regard to England, and to every other nation that has ascended from the lower to the higher levels of intellectual growth.

What is true of the whole is also true of the particular. Ask of the educated and uneducated man of the present day an opinion respecting diseases, and the way they should be met; and the position of the two systems of medicine will, in nine cases out of ten, be defined in the replies obtained. In simple trust in the value of curative measures, the uneducated man will dilate at once on the skill of the doctor of his choice, whether learned professor or avowed charlatan. In his estimation, diseases are either accidents or dispensations; of their nature he knows nothing; but he knows, when he himself is subjected to disease, which man or mode is that in which he places most confidence. In this faith he confides fully; for of what use is the doctor, unless his instructions are followed? With the educated man, again, all Esculapians are nearly the same; *i.e.*, they are but scholars who have inquired a little further than himself, and instructors in a general rather than in a particular sense. With him, the way to health is not to take this nostrum, or follow that system, but to learn why men are ill at all, whence the causes of disorder, and how these causes may be removed or prevented altogether.

These broad and opposing views are, moreover, not confined to the public merely; they extend, practically at least, into the profession of medicine itself, and are there very prominently and even dangerously represented. One physician, claiming to himself the right of being considered only as a practical man, confines his labours exclusively to curative measures. He is never tired of speaking of his cases and cures; and overburthens every scrap he publishes with a history of the numberless articles with which his prescriptions have been enriched. He, by no chance, ever remarks that he ordered the window of his patient's room to be opened, or the fire to be kept down, or the sun-light to be admitted, but states with religious accuracy any such fact as that one grain of calomel was given every four hours. Another physician, looking only at the preventive side of the question, gives up practice altogether; asserts that all diseases are preventible, throws physic to the dogs, and straightway busies his life in exploring graveyards, uprooting nuisances, compiling vital statistics, or discovering new plans of ventilation. These individuals are the prominent professional representatives of the two systems; and their different positions are thus freely delineated, not for the purpose of praising one at the expense of the other, but to bring out more forcibly what is about to be shown, that such division of thought and action is pitiful, and that the preventive and curative systems of medicine are one and the same.

In the first instance, these systems are *one* in their abstract meaning; that is to say, there is no real difference in the beginning, the end, or the objects of either. The broad and simple view of the question is, to look at absolute life and at absolute death, as at two distinct points; and at diseases of all kinds, merely as intermediate processes or steps between the one and the other. Thus observed, it becomes obvious that the removal of any disease by the removal of its cause—an act considered strictly curative—is nothing more than a continuation of that preventive act which would prohibit the cause of the disease altogether.

This is a mere truism; but one so little understood, that it is the very rock upon which the technical differences of the two systems of medicine are divided.

In the second place, the systems of prevention and of cure are each, in their separate forms, too limited for general application in the scientific treatment of disease; and this, because the questions relating to the treatment of a disorder should always have reference to the first causes of the disorder, as well as to the secondary results.

If any ground existed at all for disuniting the principles of preventive and curative medicine, it would be on the correct assumption that the body may be subjected to diseases from two sets of causes; *i.e.*, from causes external to it, and acting upon it previously in health, by interfering with one or other of its leading functions; and, secondly, from causes originating in the organism itself—the effects of certain chemical or physical modifications in its own economy.

If all diseases could be divided into two leading families based on these distinctions, it might be argued that the preventive and curative systems had each their special objects and duties. It might be said to pertain, for instance, to preventive medicine to deal with those disorders which spring out of external causes; and to curative medicine to deal simply with the second class, where the cause is in the organism itself. But, in the presence of the whole truth, this distinction or division of diseases, as arising from external and internal causes, does not hold good; since an interference with the functions of life by an external cause may not stop always at that point, but may give rise in the end to a series of systemic changes which are not removable on the removal of the cause, but persist independently and call for curative skill of the purest order.

The simplest example of this character is seen in the exposure of the body to intense cold. The first effects of such exposure are easily definable, and in most cases easily removable by the removal of the cause, which here is external in the fullest sense of the word. In the effects of cold, indeed, no more is expressed than that the body is robbed of its caloric more quickly than it can make it from its own internal resources; but the offices of caloric in the animal economy are so important, that in the absence of it the vital processes are all stopped, the muscles become rigid, the blood circulates with difficulty, and death is threatened. But granting that some preventive hand removes this cause of arrest in the functions of life, it is not necessary that health should be at once regained. In some part of the organism, where the arrest of function has been most felt, a new series of molecular changes may be instituted, ultimately requiring even surgical treatment, which of all forms is most distinctly curative.

But the broadest example of the kind now named is met with in the action of poisons received into the healthy body, of whatever kind these poisons may be, and however introduced, by the lungs, by the digestive canal, or by direct inoculation. Here the cause is strictly external, and the first act in the treatment should consist in removing the poison; but the secondary act is, in its way, distinct, and has reference to the ultimate effect of the poison on the body, apart altogether from the abnormal conditions which first gave rise to the disease.

Take the exhibition of some narcotic vapour as an instance of this nature. A man has been made to breathe a certain proportion of chloroform, and in the body of that man there is superinduced a *bonâ fide* disease which might be defined and named according to its symptoms, and which undoubtedly would be if the cause were not so obvious. But in this case, as the disorder is produced by artificial means, the symptoms receive no collective name; and the treatment, therefore, which is adopted, is simply to remove the cause, and leave the body to right itself.

But even in this simplest instance of a disease arising from a known agent, and capable of arrest by its removal, *i.e.*, by an extension of that principle of prevention which would keep the agent away from the body altogether; even in this case, removal may not lead to the perfect restoration of the patient; for here, inoccuous as the agent is, *per se*, secondary systemic mischiefs may occur which may demand a form of treatment, curative in character, and utterly disconnected from the first step in the process of cure.

If this be true as regards the effects on the body of a simple volatile substance which is at command, and which in ninety-nine cases per cent. quits the body without setting up any new molecular change, how much more true is it in the case of those organic poisons which no sooner are received into the system than they excite specific effects, leading to a virtual decomposition of the blood, to a modification of the secretions, and to structural changes in the tissues generally.

In the large family of diseases arising from causes of this nature, and including all those which have been called zymotics, the treatment should of necessity consist of a union of the preventive and curative principles. Yet these are the very diseases in which the disunion of these systems is most prominently marked: so that, when a great epidemic of these diseases breaks out, the preventives and the curatives at once take different ground.

The preventive, looking at the characters of the whole epidemic, ignores individual cases; and having formed some real or theoretical opinion as to the general cause of the disorder, thinks nothing about secondary results, but straightway modifies this water-course, or closes that burying-ground, or removes this or that nuisance, acting indeed throughout as one who, having obtained a supposed clue to the cause of all the mischief, thinks it enough to endeavour to remove this cause, leaving the actual sick to heal the sick, or else to be treated by others who do not understand the greater problems involved in the phenomena.

The curative, again, in these emergencies, forgets too commonly the leading causes of the epidemic; ignoring generalities, he looks only at individual cases. The patient is his business, and the symptoms; so the pulse is carefully counted and the character of the secretions is ascertained, and the sensations of the patient are gathered up, and eventually the treatment is based on these inquiries; so that if the circulation is quick and strong, and the skin hot and dry, there is an antiphlogistic system pursued; or in opposing circumstances, a stimulant system, and so on through a regular course of systematised treatment. In this way the grand mistake is committed of dividing causes from effects, and of proceeding as though they were distinct and unconnected phenomena.

Whether it is the greater error to omit the details of practice in reverting only to causes, or to overlook causes altogether in following out a routine of practice, based only on a knowledge of certain symptoms, and on certain formulæ supposed to possess special influences over those symptoms, is a question rather curious than important, since either system in its naked pretence is bad enough; but, judging from what we know of the past, the error has been most glaring on the side of the special curative plan. This error is now day by day becoming less; but, a century or so ago, men were so blinded by it, that, in attempting to describe an epidemic disease, they sometimes omitted the true symptoms of the disorder altogether, and unconsciously supplied the vacancy with certain other symptoms, which resulted purely from their own acts and deeds, in what they considered sound curative treatment. To prove that we do not exaggerate in making this statement, we would refer briefly to the history of an epidemic which is recorded by the distinguished Chomel as occurring in Paris in the year 1749.

The narrator commences by remarking, that on a certain date an epidemic began to seize the younger portions of the population of the worst parts of the city. In the description of the malady, sufficient is said to lead to the supposition that the symptoms were of the febrile type, with soreness of the throat; but, as great exhaustion, pallor, and convulsions, are said to have led in a few hours to the finale of the cases, the mind becomes bewildered, and is led to imagine that some new disease is offered to special consideration. It is not until the treatment comes under review, together with one or two incidental notes, that the true facts of the case are descended upon.

The treatment pursued, says Chomel, was to bleed freely at the onset; if after a few hours the fever remained, to bleed again; and finally, if anything like convulsions returned, to bleed again. The modern physiologist need not be told what were the unknown symptoms which Chomel has described. But what, after all, was the epidemic? One isolated case gives the key to the whole, though accidentally named. This was in a young lady, whom the bleeding could not subdue: or else she was not bled so freely in comparison with her age—she was seventeen. Her case, therefore, differs from the others, in that her throat was swollen as well as sore, that her skin assumed a pink colour, and that in the course of her recovery the body became swollen all over. The mystery is cleared up, in the truth that a great epidemic of *scarlet fever* was transformed by supposed curative treatment into a great and fatal epidemic of *hæmorrhage.*

*Errare humanum!* Let us review the past charitably; let us learn from its faults all that may be gathered, but not with charges too heavy on the heads of innocent offenders. There are hands yet to write our own doings, and to criticise our ignorance; and as we would that they should deal with their predecessors, so let us deal with ours.

Into such exaggerated mistakes as the one here related, we need not now fear again to fall; still the evil is not removed. When in Bermuda, a few years ago, yellow fever broke out, and the drinking water of the place, which was caught in tanks forming in almost every case friendly alliances with the cesspools, emitted an odour so execrable that the disease was by some attributed to the emanations from the water alone, the most active scientific treatment of a curative kind was carried out, the patient being meanwhile dosed with the decomposing beverage.

For many years past, the treatment of typhus has been sought after on principles purely curative; *i. e.* on principles which have their origin in the idea that certain set symptoms should be met by certain set formulæ.

Now, in regard to typhus, the sanitary teacher has long pointed out that it is the visitant of low, ill-ventilated, filthy localities, and that its main characters are those of diminished oxidation of the body; characters analogous to those arising from the gradual inhalation of a narcotic vapour. Yet so slight are the present bonds between the general and the special view of this disease, that only in these last days has the simple and obvious fact been proved by Stromeyer, that the best curative treatment of typhus is to pass pure air over the patient, in a steady and persistent current.

There are, again, many peculiar agencies, affecting whole classes of diseases, which deserve the attention of every medical practitioner, but which are often considered as belonging exclusively either to preventive or curative studies. Let one illustration bearing on this point suffice.

Writers on preventive medicines lay particular stress on the effects of temperature in producing, or rather in modifying diseases on the grand scale. They refer to the teachings of physiology, to demonstrate how thermometric influences throughout the world change and model the man. They point to the stunted Esquimaux at the pole, with his combustible provender, and slowly acting circulation. They point to the habitant of the equatorial line, with his light elastic build, and his circulation so quick that blood drawn from an artery or vein of his body is of the same colour; and they say, look at these differences! See you not that the Esquimaux, with his great chest and stomach, cannot live in the tropics, for he takes in too much fuel, and blows too hard ; nor can the equatorian live at the pole, for he has no stomach for oil, and can do but little in the respiratory department! And when they have drawn this broad outline, they follow it up, naturally enough, in regard to the effects of alternations of temperature on the body, in countries where such alternations are most marked.

But, unfortunately for this generalising spirit, the researches thus made are in themselves mere matters of interest, unless conjoined with the curative system ; an union which at this moment has little existence. Except in the use of the warm bath, the true application of which has never been fully defined, the great influence of temperature on the sick remains for the most part a sealed book. Yet, its influence on the whole universe cannot be doubted ; it is the *primum movens* of nature, the universal solvent *par excellence.* Its effects on the body, in health or disease, are most marked. Remove it faster than it is made, and the circulation is at once impeded. Arrange so that it cannot radiate freely, and the circulation doubles in its speed. In fact, in a physiological point of view, there is scarcely a symptom, or class of symptoms, incident to those two extremes of diseased action or pathology—hypinosis, evidenced in low typhus, and hyperinosis, as seen in acute sthenic inflammation—which may not be imitated by subjecting the body for long periods to extreme degrees of temperature.

As showing the influence of temperature on health, nothing more strikingly marked could be referred to than the mortality tables of the Registrar-General. "The power of cold on life varies", says Dr. Farr, "according to definite laws ; the general result being that the danger, after thirty, of dying of cold, is doubled every nine years of age ; for out of the same numbers living, to one death by cold, at the age of thirty, there are two at thirty-nine, four at forty-eight, eight at fifty-seven, sixteen at sixty-six, thirty-two at seventy-five, and sixty-four at eighty-four ; a series which represents the relative mortality by cold at these respective ages, during five weeks, amongst two millions and a half of people."

These are startling facts ; and, if they are not sufficient to arrest the attention of those who follow exclusively the curative principles of practice, let such turn to bed-side observation. Let them turn to the man under the influence of pneumonia, and watch the effect of alternations of temperature on the symptoms of that man's disorder. They will see then, if they observe dispassionately, that the influence of temperature on these, and many other diseases, is so great, that, left in the hands and at the discretion of ignorant attendants, it is often sufficient to overturn the results of all direct curative treatment, however judiciously recommended. Yet, so little is this important influence considered in the ordinary run of curative practice, that, except in the writings of the advocates of sanitary science, and of those who refer in the loosest way to the effects of warm climates on delicate or consumptive constitutions, the subject is scarcely thought worthy of notice in literature; while, practically, it is so disregarded, that in some of the hospitals of the United Kingdom a thermometer is hardly to be found.

In order, then, that the world may be benefited to the full by the learning of the physician, it is imperatively necessary that he should, in his ideas respecting the treatment of disease, no longer bind himself to one system of prevention and another of cure, but in every case consider these as one.

1. Because every disease springing from an external and preventible cause, cannot, as a general rule, be cured until such cause is ascertained and removed.

2. Because, in the majority of cases of disease arising from external sources, there is established a series of molecular changes, which continue even when the cause itself is taken away, and require distinct treatment, based on pathology and therapeutics.

3. Because, in instances where the body itself is the simple seat of the disease, the external chemical and physical influences to which the patient is subjected may materially modify the case, in regard to its progress and its leading characters.

Lastly, the union between the preventive and curative systems of medicine is demanded, for the sake of the innumerable discoveries which yet remain to be made regarding the origin of diseases. In a previous page, disorders were referred to as arising from internal and external sources; and in some broad instances this division is obvious enough. Still, our knowledge on these points goes only to show, that by far the greater proportion of diseases, of all kinds, have their origin in causes of a general and of an external character; and that when the types of disease are noted down one by one, and thus reasoned upon, it is hard to find any form or type that is not traceable, directly or indirectly, back to an external source.

This information, however, limited as it is, is peculiarly useful; since it shows, on the one hand, that no amount of research in the dead-house can, *per se*, reveal the beginning of diseases; and, on the other hand, that the most extensive inquiries into the general causes of disease, whether physical or chemical, can throw but little light on the progress of diseases, and the means by which life is destroyed, unless in such inquiries the secondary results are followed up, and the disorganisations which arise are scientifically traced out.

If the arguments in favour of the unity of Hygiene and Curative Medicine have any weight at all, they should be specially enforced now, when, by an important political measure, Medicine promises to be more decidedly divided into preventive and curative sections, and when men holding high positions in science are reckless enough to give expression to an opinion so puerile as the following, which has actually emanated from the press since the moon before last was in her apogee: "I am sorry to say that, in the performance of new duties (sanitary duties), the failures have been considerable on the parts of persons of the best promise from success in curative treatment. The qualifications for preventive service are proved to be more distinct from the qualifications for curative service, and more rare, than the medical profession and the public generally are aware of."

To deny the special fact regarding the incapacity of some who have passed from the curative into the preventive system of medicine, as referred to by this authority, would be absurd; but the dogmatic inference he therefore deduces cannot be too speedily or emphatically repudiated, as false and dangerous in principle, as founded on narrow views of the universe of life, as tending to weaken the science of medicine by dividing her sons into parties and sects, and as opposed to the wisest and broadest teachings of history.

It is an unmistakeable fact, that in every instance when a truly great master in medicine has appeared, his greatness has been based on the comprehensiveness of his views regarding life, health, disease, and death, and on the nearness with which he has approached towards some central law or unit by which these are connected or governed.

But for this spirit of comprehensive research, endorsed on each page of his writings, the labours and thoughts of Hippocrates, never more fully appreciated for their wisdom than now, had long since been forgotten; but for the influence of the same spirit, the most useful discovery of modern times had remained unseen, and Jenner had lived in vain.

